# Broccoli sprout supplementation in patients with advanced pancreatic cancer is difficult despite positive effects—results from the POUDER pilot study

**DOI:** 10.1007/s10637-019-00826-z

**Published:** 2019-06-27

**Authors:** Vladimir J. Lozanovski, Georgios Polychronidis, Wolfgang Gross, Negin Gharabaghi, Arianeb Mehrabi, Thilo Hackert, Peter Schemmer, Ingrid Herr

**Affiliations:** 1 Department of General, Visceral, and Transplant Surgery, Im Neuenheimer Feld 110, 69120 Heidelberg, Germany; 2 Section of Surgical Research, Department of General, Visceral & Transplant Surgery, University of Heidelberg, Im Neuenheimer Feld 365, 69120 Heidelberg, Germany; 3 Present Address: Division of Transplant Surgery, Department of Surgery, Medical University of Graz, Graz, Austria

**Keywords:** Broccoli sprouts, Sulforaphane, POUDER trial [NCT01879878], Pancreatic cancer, Clinical trial

## Abstract

Pancreatic ductal adenocarcinoma is a highly aggressive malignancy with short survival and limited therapeutic options. Broccoli sulforaphane is a promising new treatment due to the results of recent epidemiological, experimental and patient studies. Upon approval from the ethics committee and registration at ClinicalTrials.gov, 40 patients with palliative chemotherapy were placed into a placebo and treatment group in an unblinded fashion. Fifteen capsules with pulverized broccoli sprouts containing 90 mg/508 μmol sulforaphane and 180 mg/411 μmol glucoraphanin or methylcellulose were administered daily for up to 1 year. Twenty-nine patients were included in the treatment group and 11 patients were in the placebo group; these patients were followed for up to 1 year. The patient characteristics, overall survival and feasibility were assessed. Compared to those of the placebo group, the mean death rate was lower in the treatment group during the first 6 months after intake (day 30: 0%/18%, day 90: 0%/25%, and day 180: 25%/43%), and Kaplan-Meier analysis revealed a higher survival rate. There was a high drop-out rate (72% in the treatment group and 55% in the placebo group) after 1 year. We concluded from the Karnofsky index that the broccoli sprouts did not impact patient’s self-care and overall abilities severely. The intake of 15 capsules daily was difficult for some patients, and the broccoli sprouts sometimes increased digestive problems, nausea and emesis. We did not obtain statistically significant results (*p* = 0.291 for the endpoint at day 180), but the knowledge about the feasibility is the basis for the development of new sulforaphane drugs.

**Electronic supplementary material:**

The online version of this article (10.1007/s10637-019-00826-z) contains supplementary material, which is available to authorized users.

## Introduction

Pancreatic ductal adenocarcinoma (PDA) comprises more than 90% of all pancreatic cancer cases and is the fourth leading cause of cancer-related death [1]. At diagnosis, approximately 80% of patients have metastases due to the lack of early symptoms. Potentially curative surgical resection is limited to only a few patients, and > 90% of patients relapse and die from the disease without additional therapy [2]. In a metastatic setting, the FOLFIRINOX regimen and the nanoparticle albumin-bound (nab)-paclitaxel plus gemcitabine are standard treatment options for patients that have good performance and have shown a survival advantage compared to gemcitabine monotherapy [3]; however, PDA is notoriously resistant to treatment, and only a few patients benefit from chemotherapy.

Recent data have suggested that the regular consumption of cruciferous vegetables, such as broccoli, Brussels sprouts, cabbage, cauliflower, kale, swede and turnip, is associated with a reduced incidence of cancer [4]. The anticancer activity of these vegetables has been linked to the high content of sulfur-containing glucosinolates that vary among crucifers. The hydrolysis of the inactive glucosinolate by the plant enzyme myrosinase or by gut bacteria leads to the formation of biologically active isothiocyanates or indoles [5]. A focus has been placed on the glucosinolate glucoraphanin and its active isothiocyanate sulforaphane, which is enriched in broccoli and its sprouts [6]. Sulforaphane is one of the best studied bioactive agents from Brassicaceaes with anti-fungal, anti-bacterial, anti-viral, anti-inflammatory and anti-oxidative effects, which are combined with the ability to induce detoxifying enzymes, cell cycle arrest, and apoptosis [5]. Our studies have demonstrated that purified sulforaphane is able to overcome the pronounced therapy resistance of PDA in vitro and in vivo [7, 8], and similar results have been obtained in experimental models of prostate and breast cancer [8–11]. Epidemiological studies have suggested that the frequent intake of cruciferous vegetables might be inversely associated with the risk of developing pancreatic cancer [12–16], as well as cancer of the breast, kidney, bladder and prostate [17–22]. A Canadian study observed a significantly decreased risk of extra-prostatic manifestations of stage III and IV prostate cancer when men frequently consumed broccoli and cauliflower [17].

Several companies offer sulforaphane-rich broccoli seeds or broccoli sprout products because many patients have high hopes based on these promising data [23]. A clinical pilot trial of sulforaphane-rich broccoli sprout extracts in 20 men with recurrent prostate cancer was performed at the Oregon Health and Science University (OHSU) and the OHSU Knight Cancer Institute in Portland, Oregon, USA [24]. A daily dose of 200 μmol sulforaphane from broccoli sprout extracts was administered for 20 weeks. The effect on PSA levels was determined: one patient had a PSA decline of ≥50%, and 7 patients had smaller PSA declines that were < 50%. During sulforaphane intake, there was a significant lengthening of the PSA doubling time for all 20 patients, with an average of 9.6 months compared to 6.1 months without sulforaphane intake. The sulforaphane-rich extracts were considered safe because no grade 3 adverse events occurred. Prior studies with similar concentrations of broccoli sprout extracts confirmed that these sulforaphane concentrations are safe for patients [25–27]. A recent study in 2018 by Tahata et al. [27] evaluated comparable broccoli sprout extracts in 17 patients with atypical nevi that received 50, 100 or 200 μmol sulforaphane for 28 days. All patients completed this study and had no dose-limiting toxicities. Post-administration, the sulforaphane levels in blood increased dose-dependently. In addition, pro-inflammatory cytokines decreased, and the tumour suppressor decorin increased in patients who were administered sulforaphane compared to those who were not. These results suggest that a further dose escalation of sulforaphane is possible and that the administration of a mixture of sulforaphane and glucoraphanin may substantially increase the bioavailability of sulforaphane [23, 28]. Because the effects of sulforaphane from broccoli sprouts on the survival of cancer patients have never been studied in a prospective trial, we performed a pilot study with 40 patients suffering from advanced, surgically non-resectable pancreatic cancer who were receiving palliative chemotherapy. The patients in the treatment group were administered 15 capsules containing the powder of freeze-dried broccoli sprouts with 90 mg (508 μmol) sulforaphane plus 180 mg (411 μmol) glucoraphanin throughout the day for up to 1 year. The patients in the placebo group received the same number of identical looking capsules with methylcellulose. The patient characteristics, overall survival and feasibility were then evaluated.

## Methods

### Patients

All patients had pathologically confirmed, non-resectable, advanced PDA and were receiving palliative chemotherapy. Subjects were at least 18 years-of-age and were able to take in sufficient nutrition orally without symptoms of indigestion or problems with the passage of food, as indicated by nausea, vomiting, or the presence of a nasogastric tube. Exclusion criteria were a known intolerance to broccoli or its ingredients, impaired mental status, language problems, or the refusal of participation. All patients were recruited at the Department of General, Visceral & Transplant Surgery of the University of Heidelberg in Germany.

### Study design

This monocentric, prospective, placebo-controlled, clinical pilot study with two parallel groups (treatment and placebo) was approved by the ethics committee of the University of Heidelberg (reference No. S-347/2009). This study is registered at the ClinicalTrials.gov, a database of privately and publicly funded clinical studies, under the name “POUDER” (reference No. NCT01879878). Clinical diagnoses were established by conventional clinical and histological criteria. The study population was originally randomly divided 1:1 into a treatment group, receiving 15 capsules/day with a total of 90 mg (508 μmol) sulforaphane plus 180 mg glucoraphanin (411 μmol)/day, and a placebo group, receiving 15 identical-looking capsules filled with methylcellulose, for up to 1 year; however, due to the clear desire of many patients to be in the treatment group, we later reversed the randomization and put these patients into the treatment group. Patients were enrolled until the recruitment target of 40 study patients was reached. The randomization list was prepared by a colleague who was not involved in the medical care of the participants. Informed consent was obtained from all study participants prior to enrolment.

### Trial objectives

The principal objective of this trial was to evaluate the feasibility of administering sulforaphane-rich, freeze-dried broccoli sprouts in 15 capsules daily to patients with advanced PDA who were undergoing palliative chemotherapy, which was published as the study protocol [29]. The patients were asked to complete questionnaires and to provide the results of physical examinations; they underwent routinely performed cross-sectional imaging by computed tomography (CT) or magnetic resonance tomography (MRT), if available, at 3, 6, 9 and 12 months after the trial began. Questionnaires from the European Organization for Research and Treatment of Cancer (EORTC) and quality of life questionnaires (QLQ-PAN26, QLQ-C30) were provided. Additionally, the overall survival rate was documented.

### Sulforaphane-rich, freeze-dried broccoli sprouts

Sulforaphane was administered in the form of Deiters Broccoraphan® (gesundundkoestlich GmbH, Hamburg, Germany) with pulverized, freeze-dried broccoli sprouts packaged in capsules. Each batch of broccoli sprouts was analysed by a commercial, independent company (LADR GmbH - MVZ Dr. Kramer & Kollegen, Geesthacht, Germany) for the content of sulforaphane, its inactive precursor glucoraphanin and for the exclusion of Salmonella species. The sulforaphane content varied between 1200 mg and 1500 mg per 100 g sprout powder and contained additional glucoraphanin, by a factor that ranged from 1.75 to 2.4 times more than was expected. Broccoli sprout powder with a defined content of 6 mg (34 μmol) sulforaphane was packed per capsule, and the daily dose included 15 capsules to reach a total of 90 mg (508 μmol) sulforaphane plus approximately 180 mg (411 μmol) glucoraphanin per day. The sulforaphane in the freeze-dried broccoli sprout powder was stable based on our initial trial in which we tried to destroy the sulforaphane by autoclaving to generate the placebo capsules. However, even after more than 1 hour of autoclaving, a high amount of sulforaphane was still detectable. Therefore, we used methylcellulose as a placebo. Due to the high stability of sulforaphane, we did not give special storage recommendations to the patients. The patients were asked to take 15 capsules throughout the day, with no special instructions for how or when to take the pills, e.g., on an empty stomach or together with food; this aspect of the administration was completely unmonitored.

### Statistical analyses

This was a pilot study with 40 patients who were distributed into two groups that aimed to examine the feasibility of broccoli sprout application for patients with advanced PDA. The endpoint of interest was overall survival, and other criteria included feasibility, tumour markers, quality of life and the presence of sulforaphane metabolites in urine samples. The data are presented as single data points and as an average of the results. The standard deviations for the percentage of the patients who were deceased were calculated with a binomial distribution. For the Kaplan-Meier survival curves, the 95% confidence intervals were calculated. Statistically significant data were not expected from this pilot study, but we expected promising results that could justify a larger follow-up study.

## Results

### Patient characteristics

**Fig. 1:**
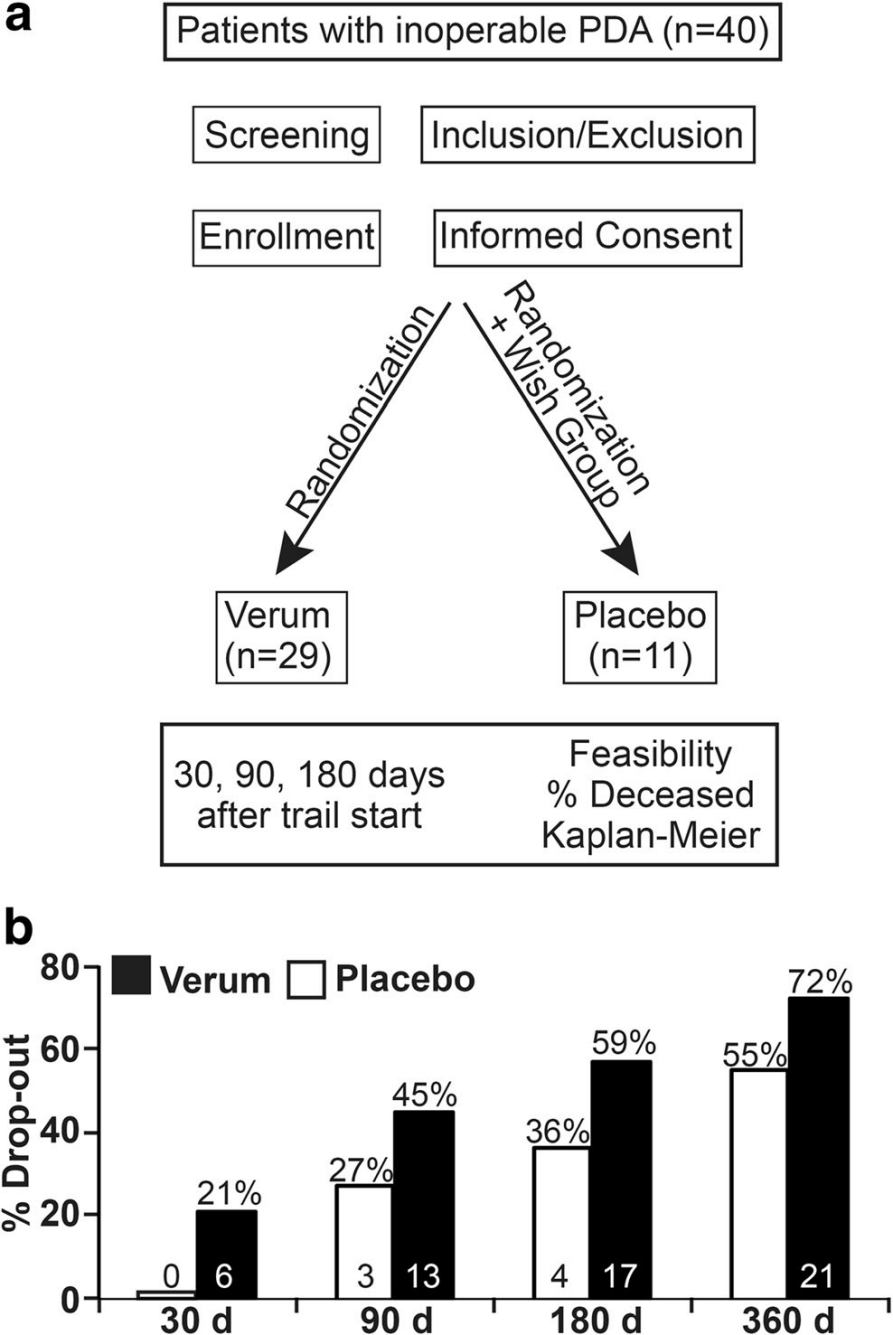
**The drop-out rate in the treatment group was higher than in the placebo group. a** Scheme showing a CONSORT diagram of the study design. **b** The diagram shows the percentage of patients who dropped out of the study before days 30, 90, 180 and 360, with an intake of 15 capsules daily of pulverized, freeze-dried broccoli sprouts in the treatment group (black bars) or methylcellulose in the placebo group (white bars). The actual number of patients at each time point is presented within the bars

This study enrolled 40 patients with inoperative PDA who were receiving palliative chemotherapy from December 2013 to October 2016; 29 patients were in the treatment group, and 11 patients were in the placebo group (see the CONSORT diagram in Fig. 1a). The most common reasons for inoperability were liver metastasis, distant metastasis, peritoneal carcinosis or infiltration of the portal vein, hepatic vein and other important veins or arteries. Originally, a double-blinded study was planned with a 1:1 distribution between the two groups; however, this strategy was not implemented because many of the patients refused to participate unless they were placed into the treatment group. Although the capsules of both groups looked identical, true blinding was not possible because the pulverized broccoli sprouts could be easily distinguished from the methylcellulose through their characteristic smell and taste. First, we tried to use inactivated broccoli sprouts as a placebo, but even several hours of autoclaving did not destroy all of the glucoraphanin and sulforaphane. Therefore, we switched to methylcellulose as a placebo. Due to difficulties in blinding the study, we offered to let the patients who wanted to be sure that they got into the treatment group choose to be in the treatment group. The patients were given a monthly supply of capsules and were asked to take 15 capsules daily to reach a total concentration of 90 mg sulforaphane and 180 mg glucoraphanin in the treatment group or methylcellulose in the placebo group. Immediately before and 3 months after inclusion in the study, patient characteristics, such as number, gender, age, body-mass index (BMI), American Society of Anesthesiologists (ASA) physical status and the Karnofsky index were determined (Table 1). Fifty-five percent of the participants in the treatment group were female and had an average age of 62 years (range 46 to 78 years) and an average BMI of 25 (range 15.5 to 39.6). Similarly, 64% of the participants in the placebo group were female and had an average age of 68 years (range 50 to 83) and an average BMI of 25 (range 20.2 to 39.6). The ASA physical status of the patients had an average score of 2.5 in both groups. The Karnofsky index was calculated with questionnaires that the patients were asked to fill out. However, only approximately one-third of the patients provided this information, and the average percentage of the available Karnofsky indices was 91% in the treatment and 87% in the placebo group at the start of the study, and the indices increased to 81% and 78% after 3 months; that represented a decrease of 11% and 10%, respectively. Overall, the group composition was comparable.

**Table 1:** Patient characteristics

Group	Patients	Gender	Age	BMI	ASA	Karnofsky		Reason inoperability	Days	Death	Drop-out
						0	3 M				
Placebo	P1	m	62	26.1	2	–	–	CVID	30	–	X
	P2	f	74	39.6	2	–	–	Infiltration *A. hepatica*	30	–	X
	P3	f	68	24.4	2	–	–	Distant metastasis	35	–	X
	P4	f	59	24.8	1	–	–	Distant metastasis	8	X	–
	P5	m	68	23.2	2	80	70	Peritoneal carcinosis/Metastasis	365	–	–
	P6	f	78	26.2	3	–	–	Infiltration VMS & A. hepatica	128	X	–
	P7	m	58	24.2	3	90	70	Liver metastasis	303	–	X
	P8	f	73	20.2	3	–	–	Liver metastasis	100	–	X
	P9	f	83	26.6	3	90	80	Infiltration VMS & V. portae	180	–	X
	P10	f	78	20.5	3	–	90	Peritoneal carcinosis	365	–	–
	P11	m	50	27.2	3	–	–	Liver metastasis	26	X	–
	**11**	**64% f**	**68**	**25**	**ø2.5**	**87%**	**78%**		**ø143**	**27%**	**55%**
Verum	V1	m	56	19.8	1	90	80	Infiltration A. hepatica	197	X	–
	V2	m	71	29.4	2	100	80	Peritoneal carcinosis	364	X	–
	V3	m	46	28.3	2	–	–	Distant metastasis	114	–	X
	V4	f	70	25.1	2	–	–	Peritoneal carcinosis/Infiltration V. linealis	17	–	X
	V5	f	61	21.0	2	80	60	Distant metastasis	163	X	–
	V6	m	63	26.6	3	–	–	Infiltration V. portae, Liver metastasis	49	–	X
	V7	m	50	21.8	3	–	–	Infiltration colon transversum	6	–	X
	V8	f	46	24.4	1	–	–	Infiltration AMS	107	X	–
	V9	m	75	15.5	3	100	90	Liver metastasis	7	–	X
	V10	f	49	19.7	2	–	–	Peritoneal carcinosis	28	–	X
	V11	f	46	27.2	3	90	80	Peritoneal carcinosis/ Infiltration coeliacus & V. portae	365	–	–
	V12	f	47	25.3	3	–	–	Peritoneal carcinosis/Liver metastasis	32	–	X
	V13	f	68	29.6	3	–	–	Liver metastasis/Infiltration duodenum	7	–	X
	V14	m	75	33.1	3	–	–	Infiltration AMS	72	–	X
	V15	f	58	28.1	2	90	80	Peritoneal carcinosis/Liver metastasis	289	X	–
	V16	f	72	23.8	3	90	90	Liver metastasis	365	–	–
	V17	m	54	26.1	3	90	90	Infiltration A. Hepatica & V. portae	180	–	X
	V18	f	78	39.6	2	90	80	Obstruction duodenum	180	–	X
	V19	f	63	23.2	3	–	–	Peritoneal carcinosis/Infiltration AMS	99	X	–
	V20	f	70	28.6	3	–	–	Peritoneal carcinosis/Infiltration AMS	5	–	X
	V21	f	74	24.4	2	–		Peritoneal carcinosis/Liver metastasis	30	–	X
	V22	m	61	24.8	3	–		Infiltration AMS & VMS	90	–	X
	V23	m	71	26.2	2	–	–	Peritoneal carcinosis	180	–	X
	V24	m	48	24.2	2	–	–	Liver metastasis	180	–	X
	V25	f	66	20.2	3	–	–	Infiltration coeliacus/Liver metastasis	87	–	X
	V26	m	72	26.6	3	–	–	Peritoneal carcinosis/Liver metastasis	30	–	X
	V27	f	57	20.5	3	–	–	Liver metastasis	100	–	X
	V28	f	73	27.2	3	90	80	Liver metastasis	90	–	X
	V29	m	62	19.8	3	–	–	Liver metastasis	30	–	X
	**29**	**55% f**	**62**	**25**	**ø2.5**	**91%**	**81%**		**ø119**	**21%**	**72%**

The bold lines indicate averages. A.: Arteria; AMS: Arteria mesenterica superior; The fitness before inclusion into the study was determined by the ASA physical status classification system and by the Karnofski Performance Status; CVID: common variable immunodeficiency; Days: Duration of participation in the study, which was finished either due to death, cancellation or reaching the maximum duration time; Death: proband died during study time; Drop-out: proband cancelled participation during the study time. Karnofsky: the Karnofsky Performance Status in percent was determined immediately before inclusion into the study (0) and 3 months after inclusion (3 M); however, some patients did not forward information about their quality of life (−). V: Vena; VMS: Vena mesenterica superior.

### Study results

The average duration of the study participation in the treatment group was 119 days, and in the placebo group, the average duration was 143 days (see Table 1). Additionally, 72% of the patients in the treatment group dropped out before the end of the planned study duration of 365 days, whereas 55% of the placebo group dropped out before the 1-year mark. Throughout the study, the percentage of patients who dropped out was higher in the treatment group compared to the placebo group. Before day 30 of the capsule intake, 21% of the patients (n = 6) in the treatment group and 0% (n = 0) of the patients in the placebo group had left the study; these numbers increased as follows: before day 90, 45% (n = 13)/27% (n = 3); before day 180, 59% (n = 17)/36% (n = 4); and before day 360, 72% (n = 21)/55% (n = 6), respectively (Fig. 1). The reasons for the discontinuation of the study were nausea, vomiting, flatulence, and other general symptoms that are associated with advanced pancreatic cancer. Some patients reported that these symptoms may have been increased as a result of the broccoli sprouts, although the decrease in the Karnofsky performance status (in the range of 11% and 10% within 3 months after inclusion) was comparable between the two groups (Table 1). In addition, many patients stopped participating in the study without providing any reason. The comparable decrease in the Karnofsky status between the two groups suggests that the broccoli sprouts did not impact patient self-care and overall abilites severely.

**Fig. 2:**
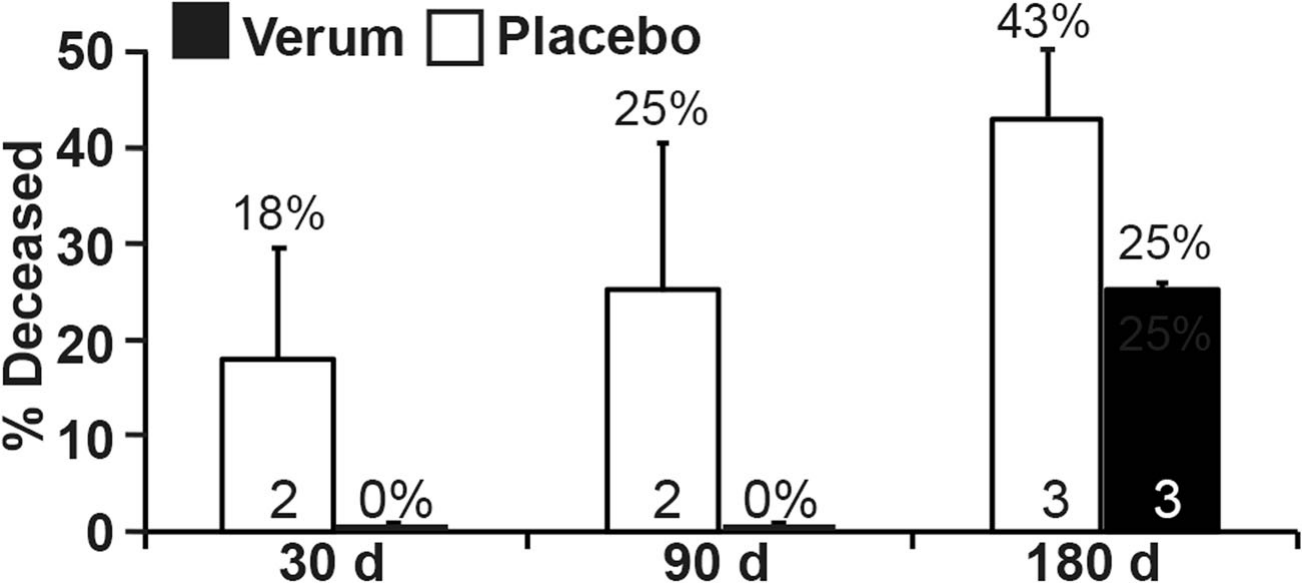
**The percentage of deceased patients was lower in the treatment group than in the placebo group.** The percentage of deceased patients in the treatment group (black bars) compared to the placebo group (white bars) is shown. Unlike Kaplan-Meier analysis, the total number of living and deceased patients was recalculated at each time point by subtracting the patients who dropped out. The actual number of patients at each time point minus those who left the study is presented within the bars. The standard deviations were calculated with a binomial distribution

Despite having a higher number of patients who dropped out of the study, within the first 180 days, the average percentage of death in the treatment group was lower than in the placebo group (Fig. 2); however, the percentage of death in the treatment group was similar to or slightly worse than that of the placebo group day 360 (Fig. S1A). However, the 1-year time point is not meaningful because only 3 patients were left in the treatment group, and 2 patients were left in the placebo group. For the calculations, we excluded the patients who discontinued the study, and we counted the dead and living patients only at a particular time point—this is different from a Kaplan-Meier analysis and was necessary due to the high rate of discontinuation. The results showed that the percentage of the patients who were deceased at day 30 in the treatment group compared to the placebo group was 0% (*n* = 0)/18% (*n* = 2); at day 90 it was 0% (*n* = 0)/25% (*n* = 2); and at day 180 it was 25% (*n* = 3)/43% (*n* = 3).

**Fig. 3:**
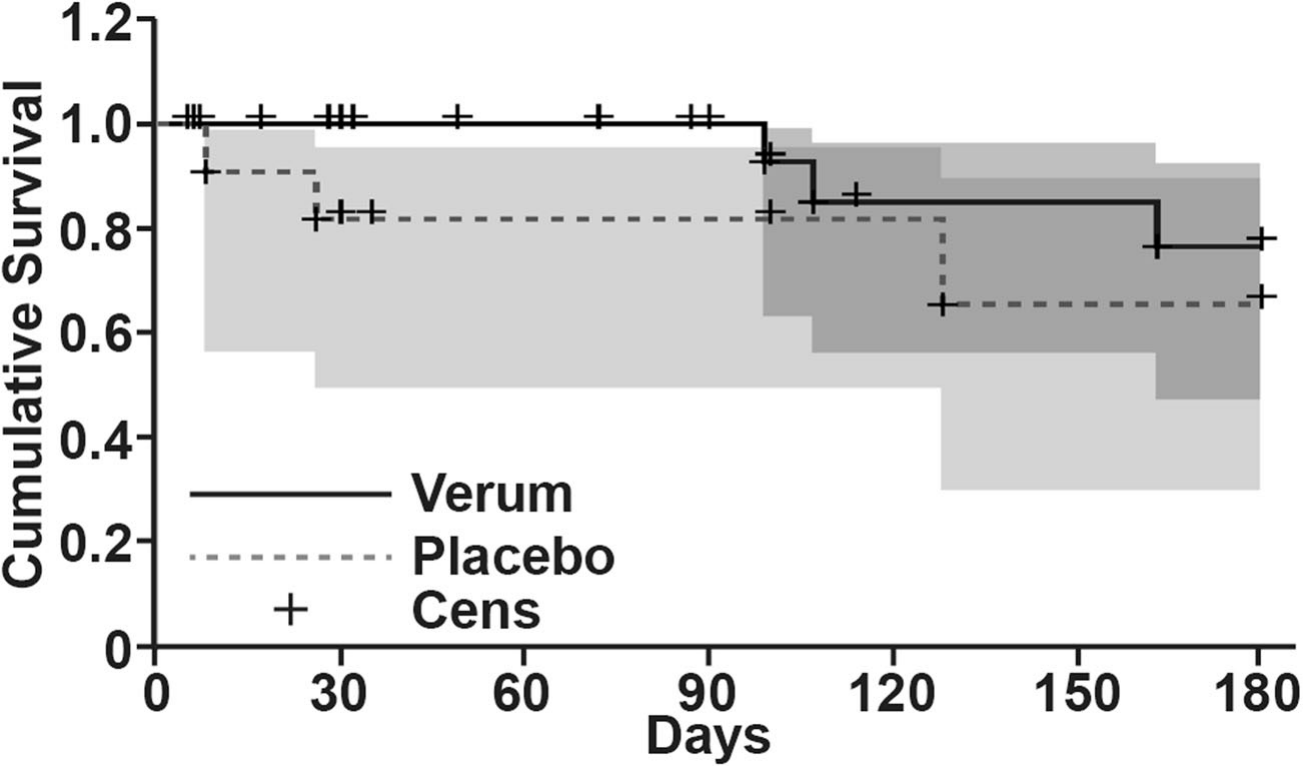
**Kaplan-Meier analysis showed a survival advantage for patients in the treatment group.** The cumulative survival over time (30 to 180 days) was calculated by setting the total number of patients in the treatment group (continuous line) and the placebo group (broken line) at the start of the study to 1. A plus sign (Cens) indicates when a patient was lost to follow-up, and a drop of the line indicates the death of a patient at a particular time point. The 95% confidence intervals for the treatment (black line) and placebo (grey, dotted line) with censors (+) are shown

Next, we performed a Kaplan-Meier survival analysis and measured the fraction of patients who lived for a certain period of time after the intake of the capsules. The period of measurement was from day 1 of the capsule intake until death. Kaplan-Meier analysis takes the study subjects that refused to remain in the study or for whom contact was lost during the study into consideration—these situations were labelled as censored observations. With this method, we observed better survival in the treatment group than in the placebo group during the first 180 days of the study (Fig. 3). At 360 days, survival decreased in the treatment group compared to the placebo group (Fig. S1B). Again, these data from late in the study are not meaningful due to the high number of patients who had left the study and the very low number of remaining patients. Although these data lack statistical significance, they are promising and may justify a follow-up trial.

## Discussion

Here, we present the results of the POUDER Trial, which was originally planned as a blinded, two-armed study. Unfortunately, blinding was not possible due to the characteristic smell of the broccoli capsules, which made them easy to identify, and because of the desire of many of the patients to be in the treatment group. We evaluated the suitability of pulverized, freeze-dried broccoli sprouts in capsules as a co-treatment in patients with non-resectable, advanced pancreatic cancer. Because the median survival at this stage is <6 months to ~11 months [3], we expected to obtain results within the scheduled 12-month treatment and observation time. Based on the extreme aggressiveness of PDA, we used a high sulforaphane dose of 90 mg (508 nmol) plus 180 mg (411 μmol) glucoraphanin, which had never been evaluated in a human study. The highest dose that had been used previously was 200 μmol sulforaphane, which was administered as broccoli sprout extract. Unlike our raw broccoli sprout preparation, the broccoli sprout extracts consisted of the aqueous phase of three-day-old, fresh, green broccoli sprouts after boiling and the conversion of glucoraphanin to active sulforaphane by daikon myrosinase after cooling [24, 27]. After digestion, the extracts were lyophilized, and 218 mg powder with 50 μmol sulforaphane was packed into capsules—the patients took 4 capsules daily [24, 27]. In contrast, our capsules had a higher volume, with approximately 400 mg powder, and had a lower sulforaphane content of 6 mg (34 μmol) but contained approximately 12 mg (27 μmol) glucoraphanin—the patients took 15 capsules throughout the day. In our study, the daily sulforaphane dose was higher, and the clearance of sulforaphane from the body was most likely slower due to the intake occurring throughout the day and the presence of additional glucoraphanin in the powder. As it was recently described, active sulforaphane could be generated from glucoraphanin by intestinal bacteria [28]. Thus, our conditions promised a substantially greater bioavailability of sulforaphane; however, the supplementation of broccoli sprout preparations in patients with advanced pancreatic cancer requires further optimization due to the following difficulties.

Although only patients who were able to take in food orally without indigestion or problems with food passage were included, patients with advanced PDA often rapidly progress from a healthy status to the terminal stage, and the progression is accompanied by a variety of severe side effects [30]. This may have been a major reason why several patients stopped participating in the study without providing a reason. The reported reasons were typical side effects of advanced pancreatic cancer, namely, pain, fatigue, lack of energy, digestive problems with a loss of appetite, taste and dry mouth, diarrhoea, constipation, abdominal pain, nausea and vomiting, sleep and neurological problems, and psychological distress [31]. These may have been the main reasons why so many patients did not complete the study. Upon the progression of their cancer, the patients were likely unable to take the required 15 capsules per day; however, there were complaints regarding the high capsule numbers from the beginning of the trial. The higher percentage of discontinuation in the treatment group may have been due to an increase in digestive problems induced by the broccoli sprouts. Additionally, disgust due to the broccoli flavour coming from the stomach after the capsule intake was reported, and this may have increased nausea and emesis; however, no severe impact on patient’s self-care and overall abilities were observed, which is highlighted by the Karnofsky performance status, which was comparable between the two groups 3 months after the study began. Our conclusion is in line with the results of former clinical phase I and II studies that demonstrated the safety and tolerance of glucosinolate- and isothiocyanate-rich broccoli sprouts in healthy volunteers and in cancer patients [6, 24, 27]; however, these studies used lower sulforaphane concentrations.

Our original intention was that only patients who were undergoing treatment at the University Clinic of Heidelberg would be included in this study because these patients were regularly on-site and could be easily followed. Despite this, nearly all cancer patients at our clinic were already included in other clinical studies, and there were concerns that participation in our powder study would influence the outcome of the other studies. We cannot rule out that the co-administered of different chemotherapeutic schedules did not affect the outcome of our study; however, patients in chemotherapy studies have no eating restrictions and can consume broccoli sprouts because it is food and not a medicament. Therefore, our pilot study was not a drug study but a dietary study. To avoid interference with ongoing drug trials in Heidelberg, we ultimately accepted patients from other clinics. These foreign patients received information via phone or e-mail and were asked to take the capsules at home. In addition, we asked these patients to complete a quality of life questionnaire and to send urine, blood and information about tumour progression at regular intervals in cooperation with their family doctor. Unfortunately, this strategy did not work well, and we lost follow-up contact with many patients. This was also the reason why we received only a small amount of blood and urine samples, which were not representative enough for analysis. As the majority of patients came from abroad, there were difficulties with direct care, and this was another major reason for the high drop-out rate. In addition, sulforaphane-rich broccoli sprout extracts are freely available, and some patients may have preferred to leave the study without informing us to take the commercially available broccoli sprout products. Regarding the low median survival rate of <6 months to ~11 months for advanced stage PDA with palliative chemotherapy [3], the documented 6 months in our study may have been a representative timeframe. At first, our study protocol intended to include urine analysis for sulforaphane metabolites to determine whether the broccoli sprouts had been consumed or if the patients in the placebo group had consumed high amounts of cruciferous vegetables. However, due to the above-described difficulties in follow-up, we did not obtain sufficient amounts of urine samples for representative tests.

## Conclusion

This study contains many important pieces of information about the feasibility of taking broccoli capsules for patients with advanced pancreatic carcinoma, but it also had limitations. For example, the Karnofsky index depends on the investigator, and we had data from only one third of the patients; thus, these data might not be representative. Additionally, we did not obtain statistically significant survival data; however, this was not expected because this was a pilot trial and due to the low number of patients and high drop-out rates. Nevertheless, we achieved our goal of obtaining promising results and new information about the feasibility of such a study in patients with advanced pancreatic cancer. Within the 180 days of follow-up, the patients in the treatment group survived longer (*P* = 0.291) than those in the placebo group. There were some drawbacks to the study, such as the drop-out rate was extremely high, 15 capsules per day were too much, and the broccoli sprouts may have increased the digestive symptoms in some patients. Therefore, the development of a new drug with a high sulforaphane content and with lower side effects is required for a future follow-up study.

## Electronic supplementary material

Fig. S1 The percentage of deceased patients was higher and the cumulative survival was lower in the treatment group at 360 days, but the data at these late time points are not meaningful due to the very low number of remaining patients in both groups. A. Comparison of the legend of Fig. 2. B. Comparison of the legend of Fig. 3. Please note that at day 360, only 3 patients were in the treatment group, and 2 patients were in the placebo group. (PDF 68 kb)

## Abbreviations

ASA: American Society of Anesthesiologists
BMI: Body-mass index
CT: Computed tomography
EORTC: European Organization for Research and Treatment of Cancer
MRT: Magnetic resonance tomography
OHSU: Oregon Health and Science University
PDA: Pancreatic ductal adenocarcinoma
QLQ: Quality of life questionnaires
