# Supplementary figures and images for: Broccoli sprout supplementation in patients with advanced pancreatic cancer is difficult despite positive effects—results from the POUDER pilot study
Source: Invest New Drugs. 2019 Jun 27;38(3):776–84. doi: 10.1007/s10637-019-00826-z (PMC7211206; doi:10.1007/s10637-019-00826-z)

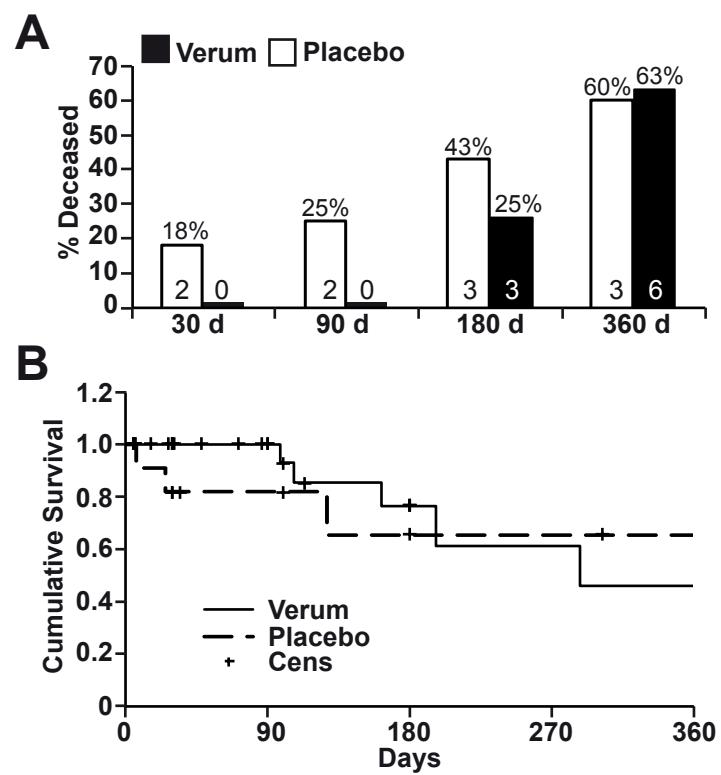

Supplement: Supplementary file 1 — The percentage of deceased patients was higher and the cumulative survival was lower in the treatment group at 360 days, but the data at these late time points are not meaningful due to the very low number of remaining patients in both groups. A. Comparison of the legend of Fig. 2. B. Comparison of the legend of Fig. 3. Please note that at day 360, only 3 patients were in the treatment group, and 2 patients were in the placebo group. (PDF 68 kb) [file 10637_2019_826_MOESM1_ESM.pdf]
